# Cellular communication network 1 promotes CASP2 mRNA expression but suppresses its protein translation in esophageal adenocarcinoma

**DOI:** 10.1002/ccs3.12046

**Published:** 2024-07-17

**Authors:** Ruize Xu, Zhenyu Jiang, Xianmei Meng, Lingling Xing, Wula Aladan, Baoxing Chi, Tong Dang, Jianyuan Chai

**Affiliations:** ^1^ Inner Mongolia Institute of Digestive Diseases Inner Mongolia Engineering Research Center for Prevention and Treatment of Digestive Diseases The Second Affiliated Hospital of Baotou Medical College Inner Mongolia University of Science and Technology Baotou China

**Keywords:** apoptosis, CASP2, CASP3, CCN1, esophageal adenocarcinoma

## Abstract

Induction of apoptosis in tumor cells is one of the best ways to cure cancer. While most apoptosis requires a chain of caspase activation, CASP2 can do this all by itself. The matricellular protein cellular communication network 1 (CCN1) is known for supporting some cancer growth but suppressing others. Esophageal adenocarcinoma (EAC) belongs to the latter. CCN1 is capable of inducing TRAIL‐mediated apoptosis in EAC cells. This study found that CCN1 upregulated CASP2 transcription but not its translation in EAC cells because, on one hand, CCN1 downregulated p16 and p21, which increased RB1 phosphorylation allowing E2F1 to transcribe more CASP2 mRNA, on the other hand, CCN1 also upregulated HuR, which is bound to CASP2 mRNA species and blocked its protein translation. As a result, CASP2 contributed nothing to CCN1‐induced EAC cell apoptosis. On the contrary, CCN1 promoted CASP3, not only in its transcription but also in its translation and activation, which established the basis for CCN1‐induced EAC cell apoptosis.

## Graphical abstract

Cellular communication network 1 (CCN1) is found to upregulate CASP2 mRNA expression but not its protein translation. Although CCN1 promotes E2F1 transcriptional activity to generate more CASP2 mRNA, it also upregulates HuR to block CASP2 protein translation. On the contrary, CCN1 promotes CASP3, not only in its transcription but also in its translation and activation, forming the basis for CCN1‐induced esophageal adenocarcinoma cell apoptosis.

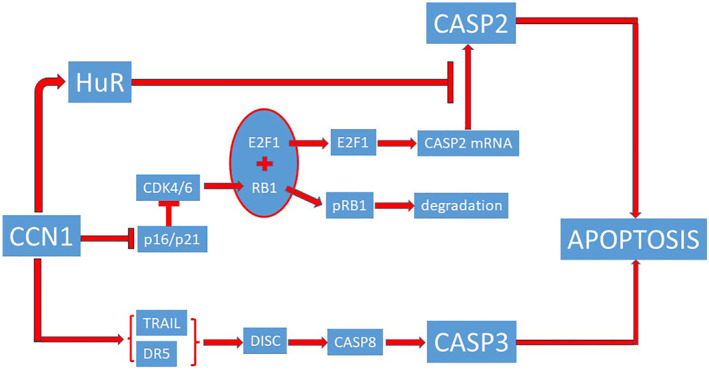

## 1 INTRODUCTION

Esophageal cancer is one of the most common malignancies in the world, what is more, its incidence is still growing year after year.^1^, ^2^ Based on a recent study,^3^ during the 30 years of 1990–2020, global cases of esophageal cancer increased by 67.1%, now becoming the eighth most common cancer worldwide. Furthermore, its mortality is as high as pancreatic cancer. Two sub‐types of esophageal cancer are commonly diagnosed: esophageal squamous cell carcinoma (ESCC) and esophageal adenocarcinoma (EAC). While ESCC has been the predominant one historically, the incidence of EAC is rising rapidly, largely due to the steady growth of gastroesophageal reflux disease (GERD). Frequent GERD episodes damage the epithelial lining of the lower esophagus, causing chronic esophagitis and columnar metaplasia. The latter can increase the odds of EAC by 400‐fold.^4^, ^5^

One of the best ways to control tumor growth is to induce apoptosis in the cancer cells selectively because apoptotic cell death does not cause cell leak and therefore no cellular content is released to the extracellular space to harm the adjacent tissue.^6^ Best of all, apoptosis takes place 20 times faster than cancer cell proliferation. Tumors would be unable to grow if apoptosis keeps happening in the tumor cells. Apoptosis can be initiated through two pathways: death receptors mediated extrinsic apoptosis and mitochondrial leak‐induced intrinsic apoptosis. Either way, it requires caspase activation. There are 12 caspases known in human, which are classified into three categories based on their distinct functions: inflammatory caspases (CASP1, CASP4, CASP5, and CASP12), apoptotic caspases (CASP2, CASP3, CASP6, CASP7, CASP8, CASP9, and CASP10), and cornification caspases (CASP14). Among the seven apoptotic caspases, CASP2, CASP8, CASP9, and CASP10 are called initiators, responsible for activating extrinsic (CASP8 and CASP10), or intrinsic (CASP2 and CASP9) apoptotic processes and awakening the executioners (CASP3, CASP6, and CASP7) to chop thousands of proteins that are essential for cell life into pieces, resulting in cell death irreversibly.^7^

Among all the apoptotic caspases, CASP2 is the most intriguing one, because it retains the features of both the initiators and the executioners. Structurally CASP2 is similar to CASP9, but it does not activate any other caspases as CASP9 does, instead, it cleaves many of the protein substrates of the executioner caspases, for example, BID, DFFA, PARP1, etc.^8^ In other words, CASP2 can initiate as well as execute apoptosis all by itself without involving CASP3, CASP6, or CASP7. It is one of the transcriptional targets of E2F1, a major regulator of cell cycle progression, whose activity is subject to RB1 (retinoblastoma) attachment and detachment. When RB1 is phosphorylated by CDK4/6 (cyclin‐dependent kinase 4/6), E2F1 leaves RB1 to go to work.^9^, ^10^

CCN1 (cellular communication network 1, or Cyr61) is a matricellular protein with a diversity of functions depending on cell type and cell stage.^11^, ^12^ It normally supports cell adhesion and migration, and thereby plays important roles in wound healing and angiogenesis.^13^, ^14^ However, it can also promote cell survival on one hand while induce cell death on the other, especially in cancer. Esophageal cancer is a great example. As we reported before,^15^, ^16^ CCN1 is overexpressed in ESCC but barely detectable in EAC. For this reason, CCN1 supports ESCC development but induces apoptosis in EAC. The current study aims to dissect the mechanisms of how CCN1 regulates CASP2 in EAC cells.

## 2 MATERIALS AND METHODS

### 2.1 Cell culture and transfection

Human esophageal cancer cells, OE19 and OE33 (Sigma‐Aldrich), were cultured in Roswell Park Memorial Institute medium plus 10% fetal bovine serum (Sigma‐Aldrich). For cell treatment, an equal number of cells were plated in 6‐well plates and cultured to 80% confluency. They were then synchronized by 6‐h starvation before incubating them with a recombinant human CCN1 protein (NM_001554, OriGene) at 1 µg/mL for an indicated time. To create an overexpression cell line for CCN1, p21, or p16, a pcDNA3.1 vector carrying an open reading frame of CCN1, CDKN1A, or CDKN2A (OriGene) was used for transfection with Lipofectamine LTX‐plus (Invitrogen); to knock down E2F1, HuR, CASP2, or CASP3, a pRS vector with shRNA specifically against each one of them (OriGene) was used for transfection. Transfection was performed using Lipofectamine LTX‐plus following the manufacturer's protocol. The control cells were transfected with either the empty vector pcDNA3.1 or pRS carrying a negative sequence (5′‐GCACTACCAGAGCTAACTCAGATAGTACT‐3′). After transfection, the cells were selected using Neomycin for pcDNA3.1 or Puromycin for pRS (Invitrogen). The transfection efficiency was monitored via Western blot analysis.

### 2.2 Real‐time RT‐PCR

Total RNA was extracted from the cells using Trizol reagent (Invitrogen) and purified using an RNeasy kit (QIAGEN). These RNA extracts were then used as templates to synthesize cDNA probes by reverse transcription using a GEArray kit (SABiosciences). The primer pairs for each interested gene were purchased from OriGene and shown in Table 1. Reverse transcription was performed following the procedure: 25°C/10 min, 55°C/30 min, 85°C/5 min, and 4°C/∞. Real‐time PCR was performed following the two‐step program using the SYBR Green master mix (SABiosciences). Data were generated from four independent experiments and analyzed according to the ΔΔ*C*_*t*_ method. Briefly, Δ*C*_*t*_ was calculated by subtracting the *C*_*t*_ value of GAPDH from the *C*_*t*_ value for each gene; and then ΔΔ*C*_*t*_ was calculated by subtracting the Δ*C*_*t*_ of the control from the Δ*C*_*t*_ of the treatment, and finally, the fold change was calculated using the formula: Fold Change = 2^(−ΔΔ*Ct*)^.

**TABLE 1:** The primer pairs (OriGene) used for RT_PCR.

Genes	Forward	Reverse
CASP2 (NM_032982)	TGCCTTCTGTGAAGCACTGAGG	CGGAAAAGGGAGACTCAAGTCG
CAPS3 (NM_004346)	GGAAGCGAATCAATGGACTCTGG	GCATCGACATCTGTACCAGACC
CASP6 (NM_001226)	AGGTGGATGCAGCCTCCGTTTA	ATGAGCCGTTCACAGTTTCCCG
CASP7 (NM_033338)	CGGAACAGACAAAGATGCCGAG	AGGCGGCATTTGTATGGTCCTC
CASP8 (NM_001081025)	AGAAGAGGGTCATCCTGGGAGA	TCAGGACTTCCTTCAAGGCTGC
CASP9 (NM_001229)	GTTTGAGGACCTTCGACCAGCT	CAACGTACCAGGAGCCACTCTT
CASP10 (NM_032977)	CCAGGCTATGTATCCTTTCGGC	TCGTTGACAGCAGTGAGGATGG
GAPDH (NM_002046)	GTCTCCTCTGACTTCAACAGCG	ACCACCCTGTTGCTGTAGCCAA

### 2.3 Northern blot analysis

A human CASP2 cDNA fragment corresponding to nucleotides 5–545 was labeled with a DIG Labeling Mix kit (Roche) following the manufacturer's protocol. Then, the labeled probe was denatured by boiling for 5 min and rapidly cooling on ice. In the meantime, 1 μg of total RNA per sample was mixed with 2 volumes of loading buffer (provided by the manufacturer) and denatured at 65°C for 10 min. The denatured RNA samples were separated in a 1% formaldehyde‐agarose gel and transferred to a Hybond nylon membrane by capillary action in high salt solution (10× standard saline citrate, 1 mM EDTA). Samples were fixed to the membrane by UV crosslinking and then prehybridized in DIG Easy Hyb at 68°C for 30 min. Precisely 100 ng/mL denatured DIG‐labeled probe was added to the membrane and incubated overnight at 68°C with gentle agitation. Blots were washed three times in 2× standard saline citrate/0.2% sodium dodecyl sulfate (SDS) at room temperature for 30 min and then in 0.5× standard saline citrate/0.2% SDS at 68°C for 2 × 15 min. The membrane was blocked and incubated in Antibody solution (provided by the manufacturer) for 30 min, and then washed and detected using CDP‐Star (provided by the manufacturer). The image was captured and quantified using Gel Doc XR (Bio‐Rad).

### 2.4 Protein isolation, IP, and Western blot analysis

After indicated cell treatment, the cellular protein was extracted using a modified radioimmunoprecipitation assay buffer lysis buffer containing 50 mM Tris–HCl (pH 7.5), 150 mM NaCl, 5 mM EDTA, 0.5% sodium deoxycholate, and 1% NP‐40. The supernatant was collected after centrifugation at 10,000*g* for 10 min.

For immunoprecipitation (IP), 100 μg of each cellular protein extract was adjusted to 1 μg/mL concentration and incubated overnight at 4°C with protein A‐agarose beads (Invitrogen) coated with 1 μg of specific antibody against RB1 (ab32513, Abcam) or HuR (ab200342, Abcam). The beads were then washed twice in the lysis buffer and boiled in the SDS sample buffer for 5 min to release the targeted protein and its associates.

Conventional Western blot analysis was performed. Briefly, equal amounts of protein samples (30–50 μg each) or IP products were separated in a mini‐gel at 100 V and transferred to a nitrocellulose membrane at 390 mA for 90 min. The membrane was first blocked with 5% nonfat milk for an hour and then incubated with a primary antibody for 2 h on a shaker. After a 3 × 10‐min wash with Tris Buffered Saline with Tween 20 buffer, the blot was incubated with an HRP‐conjugated anti‐rabbit or anti‐mouse secondary antibody (Santa Cruz Biotechnology) on a shaker for an hour. The signal was developed in Enhanced chemiluminescence solution (Amersham Pharmacia) and the image was captured and quantified using Gel Doc XR. Protein expression of interest was compared among the experimental conditions within the same blot against β‐actin. The data were collected from multiple independent experiments and assessed for statistical significance. The following antibodies were used in this study: CCN1 (TA349858, OriGene), CASP2 (ab179519, Abcam), CASP3 (TA374282, OriGene), p21 (TA808128, OriGene), p16 (ab270058, Abcam), E2F1 (TA384125, OriGene), RB1 (ab32513, Abcam), phosphorylated RB1 (ab277774, Abcam), tripartite motif 25 (TRIM25) (ab167154, Abcam), HuR (ab200342, Abcam), and β‐actin (sc69879, Santa Cruz Biotechnology).

### 2.5 IP RT_PCR

After CCN1 transfection and antibiotic selection, cells were lysed to extract protein as above. HuR was pulled down using a specific antibody (ab200342, Abcam). The mRNA species associated with HuR was extracted from the beads using the Trizol reagent. RT_PCR was performed using the primers for CASP2 (Table 1). The PCR products were separated on a 1% agarose gel containing 0.5 mg/mL ethidium bromide. Normalization of input RNA was confirmed by RT reaction of total cellular RNA isolated from the cell extract as was used for IP and subsequent assessment of GAPDH levels.

### 2.6 Apoptosis assay

Cell death was assessed using an apoptosis/necrosis detection kit (ab176749, Abcam) following the manufacturer's instruction via microscopic observation as well as flow cytometry. For microscopic analysis, cells were cultured until the desired confluence was achieved on the coverslips that had been pre‐coated with type I collagen. Briefly, cells were treated on coverslips as indicated and then incubated in the dark with a mixed staining solution containing Apopxin at room temperature for 30 min. Afterward, the coverslips were examined under an Olympus fluorescence microscope at Ex/Em 490/425 nM. Photographs were taken in 5 randomly selected microscopic areas in each one of 3 coverslips per treatment (*n* = 15), and the number of green cells was recorded. The apoptotic index (%) was calculated by dividing the number of green cells by the total number of cells in the view (verified by DAPI staining). For flow cytometry, cells were cultured in 6‐well plates and treated as for the coverslips, and then scraped off the plates and collected and re‐suspended in the assay buffer containing Apopxin. After 30‐min incubation at room temperature, the cells were analyzed in the flow cytometer (Bio‐Rad) using the same wavelengths as the microscopy.

### 2.7 Cell proliferation assay

Cell Counting Kit 8 (CCK‐8, Sigma‐Aldrich) was used to quantify cell proliferation after the indicated treatment following the manufacturer's protocol. Briefly, an equal number of cells was distributed in a 96‐well plate at 100 μL/well. The plate was incubated in the CO_2_ incubator for 24 h and then switched to the serum‐free medium for 6 h. After the indicated treatment, 10 μL of the CCK‐8 reagent was added to each well of the cell culture. The plate was then put back in the incubator for another 4 h. Cell proliferation was measured using a plate reader (Fisher Scientific) at 450 nm wavelength. The control reading was set to 100, and the rest of the readings were multiplied by 100.

### 2.8 Statistical analysis

All numerical data were expressed as mean ± standard deviation and analyzed by single classification one‐way ANOVA. In brief, the sums of squares among groups and within groups were calculated using the formula SS_among_ = sample size/2 × (mean of the treatment + mean of the control)^2^, and SS_within_ = ∑ (treatment reading − treatment mean)^2^ + ∑ (control reading − control mean)^2^, respectively. The means of squares among groups and within groups were calculated using the formula MS_among_ = SS_among_/*df*_among_ and MS_within_ = SS_within_/*df*_within_ respectively. Here *df*_among_ = number of groups − 1 and *df*_within_ = number of groups × (sample size − 1), standing for the degree of freedom among groups and within groups, respectively. The ratio of MS_among_/MS_within_ was the *F*_*s*_ value for the experiment. By referring to the *F* table, if *F*_*s*_ > *F* value in the table at *p* < 0.05, the effect of the treatment was considered significant from control.

## 3 RESULTS

### 3.1 CCN1 promotes CASP2 transcription but suppresses its translation in EAC cells

**FIGURE 1:**
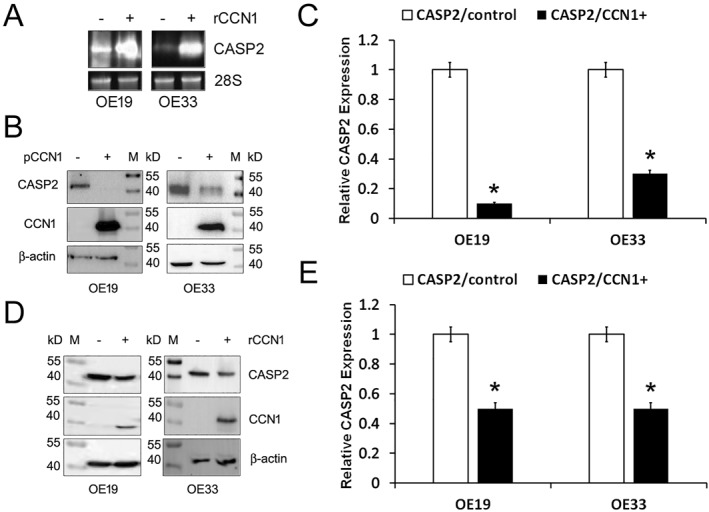
CCN1 upregulates CASP2 mRNA expression but downregulates CASP2 protein expression in EAC cells. (A) Northern blot analyses of CASP2 mRNA expression in response to treatment with rCCN1. (B) OE19 and OE33 cells were both transfected with pCCN1 or the vector. CASP2 and CCN1 expression were analyzed by Western blotting after a week of cell selection with neomycin and normalized to β‐actin. (C) The relative levels of CASP2 expression in the CCN1‐transfected cells were quantified against the vector‐transfected control, based on at least five replicates. *Statistical significance at *p* < 0.01. (D) OE19 and OE33 cells were both incubated with rCCN1 or BSA at 1 μg/mL for 6 h. CASP2 and CCN1 expression were analyzed by Western blotting and normalized to β‐actin. (E) The relative levels of CASP2 expression in the CCN1‐treated cells were quantified against the BSA‐treated control, based on at least 5 replicates. *Statistical significance at *p* < 0.01. BSA, bovine serum albumin; CCN1, cellular communication network 1; EAC, esophageal adenocarcinoma; pCCN1, pcDNA3.1‐CCN1; rCCN1, recombinant CCN1 protein.

EAC cells barely express CCN1, because CCN1 induces EAC cell apoptosis. Apoptosis depends on caspase activation. To know how CCN1 affects the caspases involved in apoptosis, we treated OE19 and OE33 cells with a recombinant CCN1 protein (rCCN1) at 1 µg/mL for 2 and 6 h. Total RNA was isolated to the examine gene expression of CASP2, CASP3, CASP6, CASP7, CASP8, CASP9, and CASP10 by quantitative RT_PCR. As shown in Table 2, CCN1 had a generally negative impact on all of the caspases except CASP2 and CASP3, especially CASP2, which was upregulated by 7052.4 ± 11.5‐ and 6341.0 ± 17.1‐fold in OE19 cells, and 5668.9 ± 20.2‐ and 19,740.2 ± 23.8‐fold in OE33 cells, after 2 and 6 h of incubation with rCCN1, respectively (*p* < 0.01). We also performed Northern blotting to confirm the RT_PCR result. As shown in Figure 1A, CCN1 treatment upregulated CASP2 mRNA expression in both OE19 and OE33 cells, consistent with the PCR experiment. Therefore, we chose CASP2 as the main focus for further investigation.

**TABLE 2:** The effect of CCN1 on caspase gene expression based on RT_PCR (fold change).

Genes	OE19/CCN1‐2 h	OE19/CCN1‐6 h	OE33/CCN1‐2 h	OE33/CCN1‐6 h
CASP2	7052.4 ± 11.5*	6341.0 ± 17.1*	5668.9 ± 20.2*	19,740.2 ± 23.8*
CASP3	71.5 ± 8.8*	133.3 ± 14.2*	1874.2 ± 17.0*	8611.4 ± 15.5*
CASP6	0.9 ± 0.2	0.7 ± 0.2	0.5 ± 0.1	1.1 ± 0.2
CASP7	0.0 ± 0.1	0.7 ± 0.2	0.8 ± 0.3	0.1 ± 0.0
CASP8	1.0 ± 0.0	0.7 ± 0.1	1.1 ± 0.3	3.7 ± 0.5
CASP9	0.0 ± 0.2	0.8 ± 0.3	0.9 ± 0.2	2.1 ± 0.8
CASP10	0.9 ± 0.3	0.8 ± 0.1	0.5 ± 0.1	0.9 ± 0.2

*Note*: OE19 and OE33 cells were treated with a recombinant CCN1 protein at 1 μg/mL for 2 or 6 h. Afterward, total RNA was isolated from the cells for real‐time RT_PCR analysis. * indicates statistical significance.

Abbreviation: CCN1, cellular communication network 1.

First of all, we transfected the cells with the plasmid pcDNA3.1‐CCN1 (pCCN1) and examined how overexpression of CCN1 affects CASP2 protein expression. To our surprise, Western blot analysis showed that CASP2 protein was not upregulated at all in response to CCN1, instead, it was almost wiped out in OE19 cells (Figure 1B,C; *p* < 0.01). In OE33, CASP2 was lowered by 75% when CCN1 was expressed. We thought this discrepancy between mRNA expression and protein expression might be due to the difference between cell transfection with a plasmid and cell treatment with a recombinant protein. Therefore, we reexamined CASP2 protein expression after 6‐h incubation of the cells with the recombinant CCN1. As shown in Figure 1D,E, CASP2 protein expression was indeed downregulated in the presence of CCN1, although the magnitude was not as great as the results from the transfection experiments, likely due to the recombinant protein decay. Anyway, the results were consistent, and both showed a negative impact of CCN1 on CASP2.

### 3.2 CCN1 facilitates the dissociation of E2F1 from RB1 by downregulating p16 and p21

**FIGURE 2:**
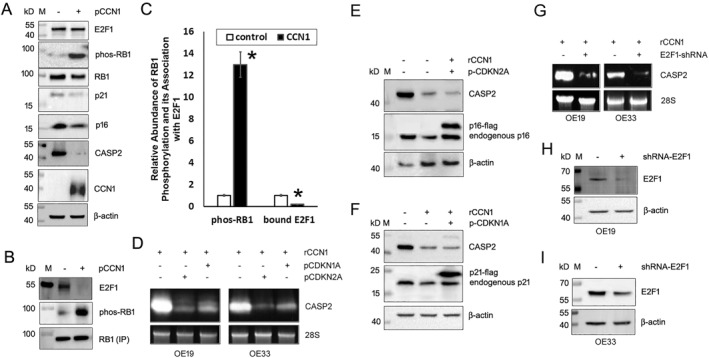
CCN1 downregulates p16 and p21 and thereby suppresses RB1 phosphorylation, allowing E2F1 to transcribe CASP2. (A) OE19 cells were transfected with pCCN1 or the vector (control) and selected with neomycin for a week. Expression of CASP2, CCN1, p16, p21, RB1, phosphorylated‐RB1, and E2F1 was analyzed by Western blotting and normalized to β‐actin. (B) RB1 was pulled down by immunoprecipitation from the transfected OE19 cells and analyzed for RB1, phosphorylated‐RB1, and E2F1 by Western blotting. (C) RB1 phosphorylation and RB1‐E2F1 binding (B) were quantified against the input protein (A), based on 3 replicates. *Statistical significance at *p* < 0.01. (D) OE19 and OE33 cells were transfected with pcDNA3.1‐CDKN1A or pcDNA3.1‐CDKN2A or the vector and selected for a week with neomycin and then incubated with rCCN1 or BSA at 1 μg/mL for 6 h. RNA was isolated and examined for CASP2 expression by Northern blot analysis. (E) CDKN2A‐transfected OE19 cells were incubated with rCCN1 or BSA at 1 μg/mL for 6 h. Protein extracts were analyzed for the expression of CASP2 and p16 by Western blotting and normalized to β‐actin. The transfected cells expressed flagged p16 along with the endogenous p16. (F) CDKN1A‐transfected OE19 cells were incubated with rCCN1 or BSA at 1 μg/mL for 6 h. Protein extracts were analyzed for the expression of CASP2 and p21 by Western blotting and normalized to β‐actin. The transfected cells expressed flagged p21 along with the endogenous p21. (G) OE19 and OE33 cells were transfected with pRS‐E2F1.shRNA (shRNA‐E2F1) or the vector and selected for a week with puromycin and then incubated with rCCN1 or BSA at 1 μg/mL for 6 h. RNA was isolated and examined for CASP2 expression by Northern blot analysis. (H) Western blot analyses for E2F1 were performed after E2F1 knockdown in OE19 cells. (I) Western blot analyses for E2F1 were performed after E2F1 knockdown in OE33 cells. BSA, bovine serum albumin; CCN1, cellular communication network 1; pCCN1, pcDNA3.1‐CCN1; rCCN1, recombinant CCN1 protein.

To resolve the discrepancy between CASP2 mRNA expression and its protein expression in the presence of CCN1, we decided to look at the whole event at the transcriptional level first. We started with E2F1, the transcription factor responsible for CASP2 gene expression. Western blot analysis showed that E2F1 protein levels were unchanged regardless of CCN1 presence or absence (Figure 2A). However, when we pulled down RB1 via IP, we found that overexpression of CCN1 not only increased RB1 phosphorylation but also reduced its association with E2F1 significantly (*p* < 0.01; Figure 2B,C), suggesting that CCN1 frees more E2F1 from RB1 control. That explains why CASP2 mRNA was upregulated by CCN1. Next, we wanted to know how CCN1 increased RB1 phosphorylation. RB1 is phosphorylated by CDK4/6, while CDK4/6 activity is repressed by cyclin‐dependent kinase inhibitors, p16 and p21. Therefore, we examined p16 and p21 expression in the CCN1‐transfected cells. As shown in Figure 2A, overexpression of CCN1 in OE19 cells downregulated both p16 and p21. Similar results were obtained with OE33 cells (data not shown). To determine whether suppression of p16 or p21 contributes to CCN1‐induced CASP2 mRNA upregulation, we forced the cells to overexpress p16 or p21 by transfection with either pcDNA3.1‐CDKN1A (i.e., p21) or pcDNA3.1‐CDKN2A (i.e., p16) plasmid, and then treated the cells with the recombinant CCN1 for 6 h. Real‐time RT‐PCR showed that overexpression of either p16 or p21 significantly attenuated CASP2 transcription that was upregulated by the presence of CCN1 otherwise (Table 3). Again, this result was confirmed by Northern blot analysis (Figure 2D). However, neither p16 nor p21 overexpression restored the CASP2 protein levels that were suppressed by CCN1 treatment (Figure 2E,F), indicating that CCN1 may interfere with CASP2 protein expression post‐transcriptionally.

**TABLE 3:** Overexpression of p16 or p21 attenuates CCN1‐upregulated CASP2 mRNA expression based on RT_PCR (fold change).

Cell types	OE19‐vector	OE19‐p16 (+)	OE19‐p21 (+)	OE33‐vector	OE33‐p16 (+)	OE33‐p21 (+)
CASP2	4311.0 ± 11.5*	15.3 ± 2.5*	22.8 ± 3.5*	18,774.8 ± 15.5*	40.2 ± 5.8*	44.4 ± 8.0*

*Note*: OE19 and OE33 cells were transfected with either pcDNA3.1‐CDKN1A (i.e., p21) or pcDNA3.1‐CDKN2A (i.e., p16) plasmid. The vector pcDNA3.1 was used as a control. After a week of selection with neomycin, all of the cells were incubated with the recombinant CCN1 protein for 6 h before the isolation of total RNA.

Abbreviation: CCN1, cellular communication network 1.

### 3.3 Knockdown of E2F1 in EAC cells blocks CASP2 mRNA induction by CCN1

There was a study showing that E2F1 negatively regulates CASP2 expression in lung carcinoma cells.^17^ To clarify whether E2F1 is a positive or negative regulator of CASP2 transcription in EAC cells, we knocked down E2F1 using shRNA plasmid specifically against E2F1 and then tested whether the recombinant CCN1 could still upregulate CASP2 transcription. As shown in Table 4 and Figure 2G, both quantitative RT_PCR and Northern blotting showed that knockdown of E2F1 significantly attenuated CASP2 mRNA expression induced by 6‐h CCN1 treatment (*p* < 0.01), suggesting that E2F1 mediates CCN1‐triggered CASP2 mRNA upregulation. Knockdown efficiency was verified by Western blotting (Figure 2H,I).

**TABLE 4:** Knockdown of E2F1 attenuates CCN1‐induced CASP2 mRNA expression based on RT_PCR (fold change).

Gene	OE19 + rCCN1	OE19/E2F1 (−) + rCCN1	OE33 + rCCN1	OE33/E2F1 (−) + rCCN1
CASP2	6341.0 ± 17.1*	4.0 ± 1.5*	19,740.2 ± 23.8*	2.2 ± 0.8*

*Note*: OE19 and OE33 cells were transfected with pRS‐E2F1.shRNA or the vector. After a week of selection with puromycin, all of the cells were incubated with the recombinant CCN1 protein for 6 h before the isolation of total RNA. * indicates statistical significance.

Abbreviations: CCN1, cellular communication network 1; rCCN1, recombinant CCN1 protein.

### 3.4 CCN1 suppresses CASP2 translation by upregulating HuR

**FIGURE 3:**
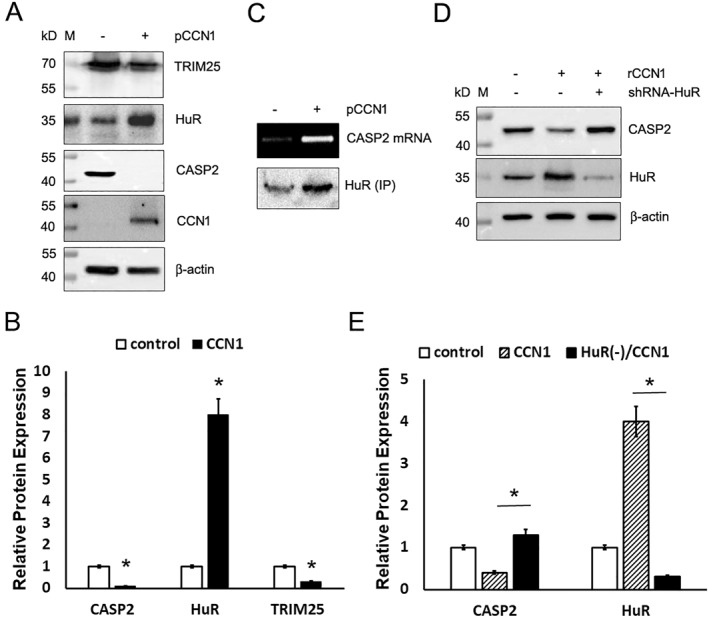
CCN1 downregulates CASP2 protein expression by increasing HuR expression. (A) OE19 cells were transfected with pCCN1 or the vector and selected for a week with neomycin and analyzed for expression of TRIM25, HuR, CASP2, and CCN1 and normalized to β‐actin. (B) TRIM25, HuR, and CASP2 expression in CCN1‐transfected cells were quantified against the vector‐transfected cells (control). *Statistical significance at *p* < 0.01. (C) HuR was pulled down from an equal amount of protein extracts of the transfected cells by immunoprecipitation and analyzed by either Western blotting for HuR (lower panel) or RT_PCR for CASP2 mRNA (upper panel). (D) HuR expression in OE19 cells was knocked down using specific shRNA against HuR and selected for a week with puromycin and then incubated with rCCN1 or BSA at 1 μg/mL for 6 h. The protein extracts were analyzed for expression of CASP2 and HuR by Western blotting and normalized to β‐actin. (E) CASP2 and HuR expression were quantified against to control. *Statistical significance at *p* < 0.01. BSA, bovine serum albumin; CCN1, cellular communication network 1; pCCN1, pcDNA3.1‐CCN1; rCCN1, recombinant CCN1 protein.

Based on the experiments above, there was no doubt that CCN1 promotes CASP2 transcription, which means that the CCN1‐induced reduction of CASP2 protein expression happens at the translational level. There were several proteins reported to inhibit CASP2 translation, typically TRIM25 and ELAV1‐like protein (HuR), both can bind to CASP2 mRNA blocking its translation. Therefore, we examined how CCN1 affects the expression of these two molecules. As shown in Figure 3A,B, CCN1 downregulated TRIM25 as it did to CASP2, but upregulated HuR. Then we decided to look at HuR further. We pulled down HuR by IP, extracted RNA from the precipitants, and ran RT_PCR using the specific primers for CASP2 (Table 1). As shown in Figure 3C (upper panel), overexpression of CCN1 increased the amount of CASP2 mRNA associated with HuR protein. HuR IP was confirmed by Western blotting (Figure 3C, lower panel), which also demonstrated an increase of HuR protein in response to CCN1 transfection.

To confirm that HuR was responsible for CCN1‐induced downregulation of CASP2 protein expression, we knocked HuR down in OE19 cells using shRNA transfection and then treated the cells with the rCCN1 for 6 h. As shown in Figure 3D,E, the knockdown of HuR restored CASP2 protein levels despite the presence of CCN1.

### 3.5 CCN1 induces EAC cell apoptosis with or without CASP2

**FIGURE 4:**
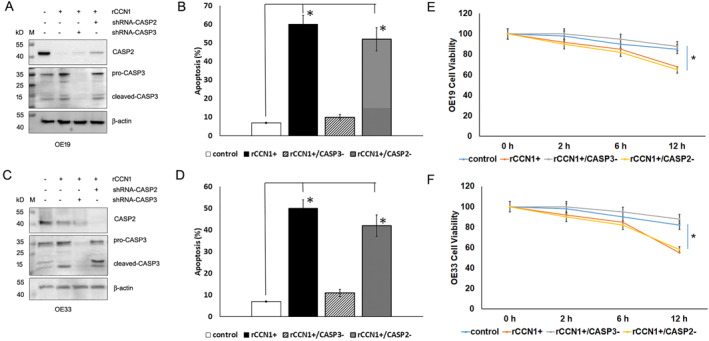
CASP3, not CASP2 mediates CCN1‐induced EAC cell apoptosis. (A) OE19 cells were transfected with specific shRNA to knock down CASP2 or CASP3 and selected for a week with puromycin, and then treated with rCCN1 for 6 h. Expression of CASP2 and CASP3 were analyzed by Western blotting and normalized to β‐actin. (B) Apoptosis in the transfected OE19 cells was assessed using Apopxin under rCCN1 treatment compared to BSA (control), based on five independent experiments. *Statistical significance at *p* < 0.01. (C) OE33 cells were transfected with specific shRNA to knock down CASP2 or CASP3 and selected for a week with puromycin, and then treated with rCCN1 for 6 h. Expression of CASP2 and CASP3 were analyzed by Western blotting and normalized to β‐actin. (D) Apoptosis in the transfected OE33 cells was assessed using Apopxin under rCCN1 treatment compared to BSA (control), based on five independent experiments. *Statistical significance at *p* < 0.01. (E) OE19 cells were transfected with specific shRNA to knock down CASP2 or CASP3 and selected for a week with puromycin. Then cells were treated with rCCN1 for 0, 2, 6, or 12 h and analyzed for cell proliferation using a CCK‐8 kit. (F) OE33 cells were transfected with specific shRNA to knock down CASP2 or CASP3 and selected for a week with puromycin. Then cells were treated with rCCN1 for 0, 2, 6, or 12 h and analyzed for cell proliferation using a CCK‐8 kit. BSA, bovine serum albumin; CCN1, cellular communication network 1; EAC, esophageal adenocarcinoma; rCCN1, recombinant CCN1 protein.

From our previous study,^15^ we learned that CCN1 induces EAC cell death through TRAIL‐mediated extrinsic apoptosis, which suggests that CCN1 must activate executioner caspases in EAC cells. In agreement with this expectation, our RT_PCR experiment above (Table 2) did show CASP3 upregulation in response to CCN treatment. To distinct the role of CASP2 from CASP3 in CCN1‐induced EAC cell apoptosis, we transfected cells with shRNA specifically against CASP2 or CASP3 and then treated the cells with recombinant CCN1. Apoptotic cell death was assessed using the Apopxin reagent. It turned out that the knockdown of CASP2 did not affect CCN1‐induced apoptosis in either OE19 (Figure 4A,B) or OE33 cells (Figure 4C,D), but the knockdown of CASP3 did (Figure 4A–D). Moreover, Western blot analysis demonstrated that CCN1 not only upregulated CASP3 protein expression but also increased its cleavage, indicating that CCN1 requires CASP3 but not CASP2 to induce EAC cell apoptosis. In agreement with the apoptosis analysis, cell proliferation assay also showed that EAC cell viability was reduced in the presence of CCN1; the lack of CASP2 did not release the cells from this pressure but CASP3 deficiency did (Figure 4E,F).

## 4 DISCUSSION

In summary, this study finds that although CCN1 promotes CASP2 transcription dramatically, this upregulation of mRNA does not turn into CASP2 protein, instead, it results in a reduction of CASP2 protein, because CCN1 upregulates HuR, which blocks the translational process of CASP2 mRNA. However, CCN1 also promotes CASP3 transcription, which leads to protein upregulation and activation. Taking together with our previous study,^15^ CCN1 induces EAC cell death through TRAIL‐mediated extrinsic apoptosis with CASP3 as the executioner, without CASP2 involvement.

We do not know why CCN1 upregulates CASP2 transcription but does not turn it into a functional protein. Here is one of our guesses. To become a functional caspase, CASP2 needs PIDD1 (p53‐induced death domain protein) to build a platform called PIDDosome for it,^18^ which contains a specific fragment of PIDD1 (100 kD) called PIDD‐CC (37 kD) and an adaptor protein named CRADD (CASP2‐ and RIPK1‐domain‐containing adaptor with death domain). After synthesis, PIDD1 is constitutively cleaved into two fragments: PIDD‐N (48 kD) and PIDD‐C (51 kD). The latter can translocate into the nucleus to form a complex with RIPK1 (receptor‐interacting serine/threonine kinase 1) and NEMO (NFκB kinase subunit γ). NEMO brings the complex to the cytoplasm, where it degrades IκBα (NFκB inhibitory protein α), leading to NFκB activation, which supports cell growth primarily.^19^ However, PIDD‐C can be further cleaved into PIDD‐CC upon severe DNA damage.^20^ Therefore, during the construction process of a PIDDosome, there is a chance for NFκB activation, which may favor tumor growth. CCN1 would not take the chance when it aims to eliminate the cell. Secondly, although an active CASP2 can cleave BID to initiate intrinsic apoptosis, it is a lot less efficient compared to CASP8. In EAC cells, CCN1 upregulates TRAIL and its death receptor DR5,^15^, ^16^ which constitute a death‐inducing signaling complex (DISC) to activate CASP8. To cleave BID, CASP2 participation seems unnecessary when CASP8 is activated. Furthermore, although CASP2 can cleave DFFA to activate DFFB, resulting in DNA fragmentation, a hallmark of apoptosis, CASP3 can do it too and is even much more powerful. Why does it need CASP2 for while CASP3 is on board?

An E2F1 binding site is located in the promoter region of the CASP2 gene,^21^ but there are conflicting reports on its role in the transcriptional regulation of CASP2. While some studies showed a positive regulation of CASP2 by E2F1,^10^ one study found that knockdown of E2F1 in lung carcinoma cells (H1299) upregulated CASP2 protein expression.^17^ This study also found that the knockdown of p21 had a similar effect on CASP2 as the knockdown of E2F1 did. These results raise a question. While p21 is well known for interfering with RB1 phosphorylation and thereby suppressing E2F1 activity, how can the knockdown of E2F1 achieve the same effect on CASP2 as the knockdown of p21? Both E2F1 and p21 work at the upstream of CASP2 transcription, therefore, we think, CASP2 mRNA expression in response to either E2F1 or p21 inactivation should have been examined. The rise of CASP2 protein expression seen in this study after E2F1 knockdown could be caused by other factors coincidently associated with E2F1, and if so, calling E2F1 a repressor of CASP2 is a little bit too casual, we would say. Several proteins are known to be capable of interfering with CASP2 translation, such as TRIM25,^22^ HuR,^23^ and BCL9L.^24^ If E2F1 knockdown interfered with the function of any of these proteins, it could lead to the rise of CASP2 level.

The role of CCN1 varies dramatically in different cell systems. It has been found to support tumor growth in breast cancer,^25^ ovarian carcinoma,^26^ and pancreatic carcinoma,^27^ but suppress tumor growth in non‐small cell lung cancer,^28^ endometrial adenocarcinoma,^29^ and hepatocyte carcinoma.^30^ In esophageal cancer, we found that CCN1 promotes ESCC^16^ while is lethal to EAC.^15^ Its impact on other molecules also varies from cell to cell. For instance, between CCN1 and p21, CCN1 has been found to upregulate p21 in trophoblast,^31^ osteosarcoma,^32^ hepatocyte carcinoma,^33^ and lung cancer,^28^ but downregulate it in prostate cancer^34^ and ovarian cancer.^35^ In EAC cells, we found that CCN1 downregulates p21. p21 is one of the transcriptional targets of p53, which is mutated but still functional in OE19 cells.^36^ Upon p53 activation, p21 is upregulated in the nucleus to suppress E2F1 by inhibiting RB1 phosphorylation, leading to cell cycle arrest, while some p21 moves to the cytoplasm where it binds the executioner caspases to prevent their activation and apoptosis.^37^ Therefore, downregulation of p21 is another way for CCN1 to facilitate EAC cell apoptosis.

## AUTHOR CONTRIBUTIONS

Ruize Xu and Zhenyu Jiang conducted most of the experimental work, prepared the data, and drafted the manuscript. Lingling Xing, Wula Aladan, and Baoxing Chi participated in experimental work and data collection. Xianmei Meng and Tong Dang provided the resources and funding and participated in data analyses and project management. Jianyuan Chai conceptualized the study, curated the results, and finalized the manuscript.

## CONFLICT OF INTEREST STATEMENT

The authors declare no conflicts of interest.

## ETHICS STATEMENT

The study was approved by the R&D Committee of Baotou Medical College. No human or animal subject was used.

## Data Availability

Data available upon reasonable request.
